# Potential targets of natural medicines: preventing lung cancer pre-metastatic niche formation by regulating exosomes

**DOI:** 10.3389/fonc.2023.1137007

**Published:** 2023-08-28

**Authors:** Xiao-yu Zhu, Jie Li

**Affiliations:** Department of Oncology, Guang’anmen Hospital, China Academy of Chinese Medical Sciences, Beijing, China

**Keywords:** lung cancer, pre-metastatic niche, natural medicines, exosomes, anti-cancer

## Abstract

Lung cancer is one of the most devastating diseases worldwide with high incidence and mortality, and the incidence continues to rise. Metastasis is the leading cause of death in lung cancer patients, yet the molecular effectors underlying tumor dissemination remain poorly defined. Research findings in recent years confirmed primed microenvironment of future metastatic sites, called the pre‐metastatic niche, is a prerequisite for overt metastasis. Exosomes have recently emerged as important players in pre‐metastatic niche formation. Natural medicines have traditionally been rich sources of drug discovery. Some of them exhibit favorable anti-lung cancer activity. The review focused on the latest advances in the regulation of the pre‐metastatic niche formation in lung cancer by the contents of exosomes of representative natural medicines. Additionally, the mechanism of natural medicines was summarized in detail, which would provide new insights for anti-cancer new drug development.

## 1 Introduction

Lung cancer is one of the most prevalent malignancies worldwide, with recent studies showing that more than 2 million people are estimated to be newly diagnosed with lung cancer each year (1), with approximately 236,740 new lung cancer diagnoses and 130,180 deaths in the United States in 2022 (2), and an estimated 820,000 new lung cancer diagnoses and 715,000 lung cancer-related deaths in China in 2020 (3). Lung cancer remains the most common cancer in China today (4). Metastasis is the leading cause of death in lung cancer patients (5), and when present, it usually predicts a poor outcome and is a malignant event that is strongly avoided in clinical practice. Metastasis may be caused by a number of factors, such as the invasive ability of cancer cells, the absence or suppression of immune surveillance function, etc. However, regardless of the mechanism of metastasis, the final manifestation is that the detached cancer cells arrive at a new proliferation area and develop into new tumor tissue. During this process, the area where the tumor colonizes forms a pre-metastatic niche (PMN) (6). The characteristics of the pre-metastatic ecotone include inflammation formation, enhanced angiogenesis and vascular permeability, lymphangiogenesis, immunosuppression, and metabolic reprogramming, and possesses four stages of initiation, licensing, colonization, and progression (7). The reasons for how PMN formation begins before tumor cells arrive are unclear, and tumor-associated exosomes appear to be good mediators of this remote regulation. Exosomes from highly metastatic cancer cells and advanced lung cancer sera have been found to induce migration, invasion and proliferation of non-cancerous recipient cells in the clinic (8), and more basic experiments likewise support the involvement of tumor-derived exosomes in cancer metastasis through multiple mechanisms by remodeling the tumor microenvironment (9). As research continues, new studies have demonstrated the ability of exosomes to seed tumor cells to appropriate ecological niches for the proliferation and formation of “micro-metastases” (10). Having clarified that the involvement of exosomes in inducing pre-metastatic ecological niches is the central mechanism of metastasis occurrence, the search for key interventions is necessary. Natural medicines are a large class of uncommercialized components derived from nature, including some natural compounds of plant origin, herbal medicines and their mixed compositions (i.e., traditional Chinese medicinal preparation) that have been empirically applied (11). They have good effects in the treatment of malignant tumors such as lung cancer (12), but the exosome-related research has not yet become systematic and deserves further exploration. In this paper, we analyze the potential natural medicines intervention targets of each component in PMN formation, and explore the novel antitumor modalities such as natural medicines that can modulate the release of exosomes and natural medicines that artificially encapsulate exosomes and plant-derived exosomes, supporting the clinical challenge that natural medicines can be involved in exosome-mediated formation of pre-metastatic ecological sites in lung cancer.

## 2 Exosome involvement in lung cancer progression and metastasis

Exosomes are a subtype of extracellular vesicles (EV), a lipid bilayer enclosed vesicle with a diameter of 30-100 nm, and one of the important mediators of intercellular communication (13). Exosomes can be secreted by various cells and can also carry a variety of substances such as lipids, nucleic acids, and proteins, and are widely distributed in body fluids, and this special property leads to their important role in information transfer in tumor metastasis (9). The role of exosomes is mainly determined by their contents, which include proteins, lipids, enzymes, transcription factors, DNA fragments, messenger RNA (mRNA), micro RNA (miRNA), and long non-coding RNA (lncRNA), etc (14). Currently, RNA, DNA and proteins are the most studied ones. Tumor cells can transfer these molecules into stromal cells to ensure communication in the microenvironment and can modify the recipient cell phenotype to be tumorigenic, participating in processes such as induction of angiogenesis, tumor cell migration and proliferation, inflammatory response, immunosuppression, evasion of immune surveillance, and metastasis (14). It has also been demonstrated in clinical settings that exosomes are more abundant in circulating body fluids of lung cancer patients than healthy individuals, and these exosomes can similarly promote the formation of lung cancer microenvironment, increase tumor metastasis, mediate tumor immunosuppression, and participate in radiotherapy resistance in ways that promote lung cancer development (15). miRNA is one of the most prominent components of exosome loading, and several miRNAs have been obtained to promote lung cancer metastasis supported by evidence, such as miR-17-92, several miR-200 family miRNAs (miR-200a, miR-200b, miR-200c, miR-141 and miR-429) including miR-125a-3p/5p, miR-21 and miR-106b-25 family (miR-106b and miR-93), among others (16). Although it is confirmed that exosomes can promote cancer metastasis, the specific mechanisms by which they exert their effects remain complex, and the existing scientific hypotheses are specifically as follows: (1) exosomes from tumor cells induce epithelial mesenchymal transformation and degrade the stroma, such as prostate cancer; (2) tumor-secreted exosomes derived from prostate cancer interfere directly or indirectly with endothelial cells by activating macrophages; (3) circulating tumor cells (CTCs) derived from leukemia, lymphoma and breast cancer and tumors activated platelets derived from lung cancer release exosomes that affect immune cells; (4) exosomes attached to tumors upregulate adhesion molecules on endothelial cells, such as chronic myelogenous leukemia; (5) exosomes can disseminate tumor cells to suitable ecological sites for proliferation and form micro-metastases, such as pancreatic adenocarcinoma and melanoma (10). In this process, the formation of pre-metastatic ecological sites as a direct manifestation of “pre-metastasis”, probably exosomes plays a more specific role.

## 3 Exosomes are involved in constructing the pre-metastatic microenvironment from multiple perspectives

**Figure 1:**
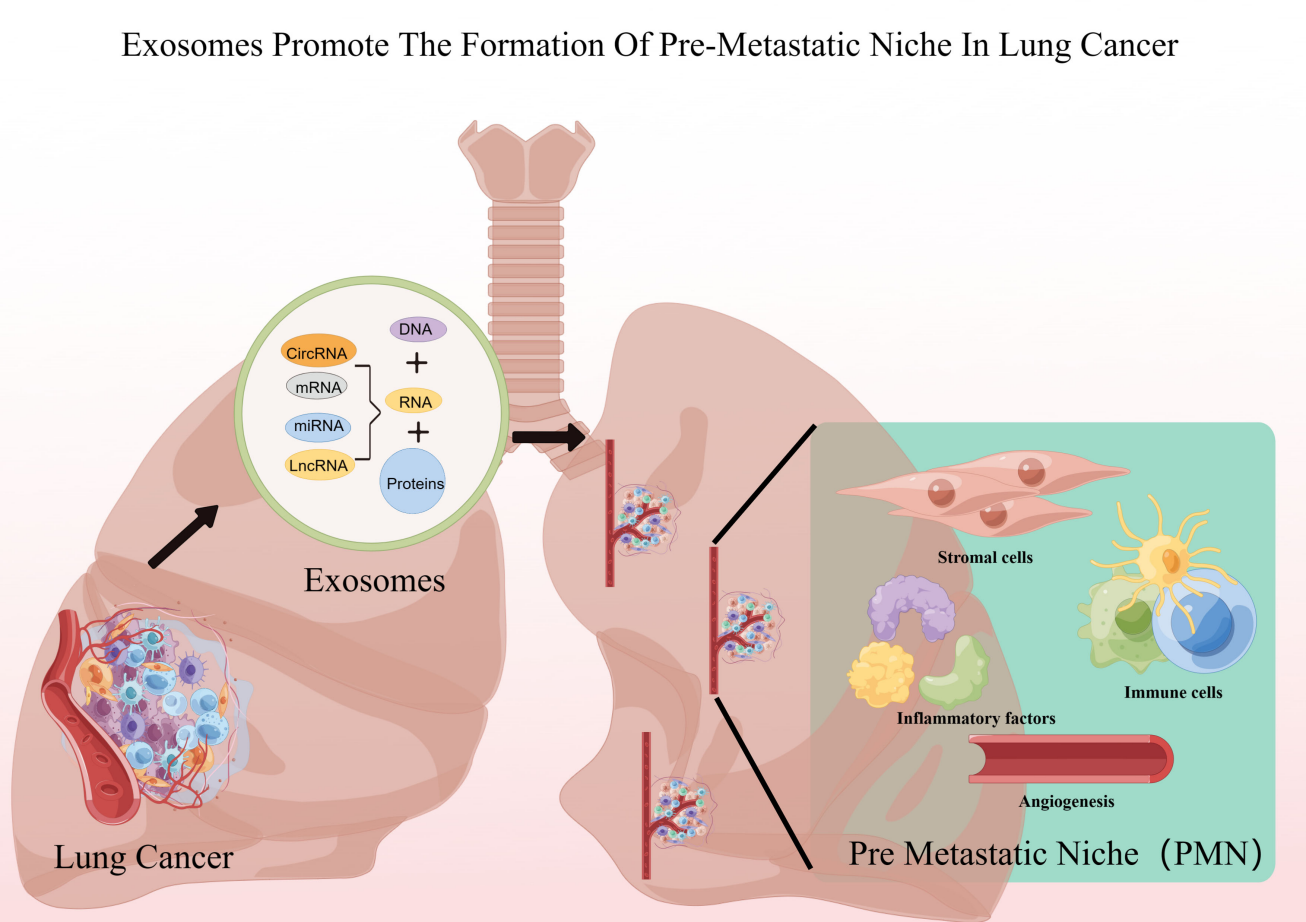
Lung cancer exosomes promote PMN formation. Figure was created by Figdraw (www.figdraw.com).

Exosomes play a crucial role in PMN formation (17). Previous studies have demonstrated that exosomes play different roles in each of the sequential steps necessary to complete the construction of the pre-metastatic ecotone (i.e., angiogenesis and leakage, PMN stromal cell domestication, bone marrow-derived cell domestication and recruitment) (18), and others recognize that PMN formation is preceded by exosomes through the induction of an inflammatory response, which may be the true onset of PMN formation (19) (**Figure 1**; **Table 1**).

**Table 1:** The role of exosomes in constructing PMN.

No	PMN formation	Exosome composition	Related pathway	Function	Refs
1	Inflammatory reaction	HSP70	TLR2/NF-κB	IL-6, IL-8, MCP-1	(20)
		U1 snRNA	TLR2/NF-κB/MAPK	promoting neutrophil recruitment	(21)
		miR-21/ miR-29a	TLR7/TLR8/NF-κB	TNF-α,IL-6	(22)
2	Angiogenesis	CEMIP	Wnt signaling	Tnf, Ptgs2, Ccl/Cxcl	(23)
		LRG1	TGF-β signaling	VEGFA, Ang1	(24)
		miR-23a	HIF-1 α/ZO-1	VEGF	(25)
		miR-21	STAT3	VEGF	(26)
3	Stromal cell domestication	miR-210	TIMP-1/PI3K/AKT	HUVECs	(27)
		GPX10	HIF-1α	epithelial cells	(28)
		miR-3473b	NF-κB	fibroblasts	(29)
4	Bone marrow-derived cell domestication and recruitment	miR-21	PI3K/AKT	M2 TAM	(30)
		TRIM59	NLRP3	macrophage	(31)
		EGFR	IDO	Treg	(32)
		exosomes	CCR6/CCR7/CXCR3	DC	(33)
		PD-L1	PD-L1/PD-1	CD8 T cell	(34)
		PD-L1	IFN-γ	CD8 T cell	(35)
5	Other factors	linoleic acid	TLR2/NF-κB	metabolic reprogramming	(36)
		exosomes from LLC	adipocytes	lipolysis	(37)
		miR-26a	Wnt	EMT	(38)
		SNAI1	E-cadherin	EMT	(39)
		TRAF6	PM/NF-κB	promoting neutrophil recruitment	(40)

### 3.1 Inflammatory reaction

Mild inflammatory response is a favorable condition for lung cancer metastasis (41), but it is worth exploring how to precisely create a suitable PMN environment for pre-metastatic cancer cells. It has been pointed out that the metastasis of tumor cells to target organs is not random, and exosomes expressing integral proteins carrying integrins α6β4 and α6β1 on the surface of exosomes are more inclined to lung fibroblasts and epithelial cells, and tumors are prone to metastasis to the lung (42), therefore, locating after suitable PMN location, the inflammatory response begins to occur. Lung cancer cell-derived exosomes can activate Toll-like receptor 2 (TLR2)/NF-κB signaling *via* their surface heat shock protein 70, converting naïve MSCs in PMN into a new pro-inflammatory MSC that secretes IL-6, IL-8 and human monocyte chemotactic protein-1(MCP-1) to maintain a low-grade inflammatory microenvironment (20). Liu et al. found that lung epithelial cells with Toll-like receptor 3 (TLR3) deficiency prevented ecotone formation, whereas exosomal RNA reversed this process by activating TLR3, NF-κB and MAPK thereby recruiting neutrophils to maintain the inflammatory environment and facilitating ecotone formation (21). Interestingly, the TLR family seems to be quite importantly present in the inflammatory response prior to PMN formation, not only TLR2 as well as TLR3 above, but also other studies have confirmed that exosomal miR-21 and miR-29a secreted by lung cancer cells can bind to mouse TLR7 (Toll-like receptor 7) and human TLR8 (Toll-like receptor 8) in immune cells, inducing TLR-mediated inflammatory responses, which may eventually provide the right conditions for tumor metastasis (22). Of course, some other inflammation-related factors beyond TLR are also of interest, such as studies confirming that transcripts of TGF-β, lnc-MMP2-2, and IL-10 in lung cancer cell exosomes play a key role in regulating the migratory ability of lung cancer cells by targeting and regulating related genes (9).

### 3.2 Angiogenesis

Inflammation may be a key factor in the induction of tumor angiogenesis and may likewise be involved in angiogenesis in PMN formation (43). The involvement of tumor-derived exosomes in the induction of inflammatory PMN formation in distant organs to promote metastasis has been supported, as cell migration-inducing and hyaluronan binding protein (CEMIP) of lung tumor origin can induce pro-inflammatory vascular ecotopes to promote metastasis by upregulating cytokines prostaglandin endoperoxide synthase 2(PTGS2), tumor necrosis factor(TNF) and chemokine (C-C Motif) ligand(CCL)/chemokine (C-X-C motif) ligand(CXCL) cytokines (23). Also mediated by inflammatory factors, NSCLC cells use exosomes loaded with LRG1 and promote angiogenesis through TGF-β pathway-mediated expression of pro-angiogenic markers (e.g. VEGFA and Ang1) (24). Hypoxic environment is also a condition for induction of angiogenesis, as exosomal miR-23a of lung cancer cell origin directly inhibits prolyl hydroxylases PHD1 and PHD2, leading to accumulation of hypoxia-inducible factor-1 (HIF-1) in endothelial cells to enhance angiogenesis (25). In lung cancer, not only the exosomes of cancer cells can induce angiogenesis, but also bronchial epithelial cells under smoking induction. For example, bronchial epithelial cells under smoke intervention elevated exosome miR-21 levels, and these miR-21 led to STAT3 activation, which increased VEGF levels in receptor cells and promoted angiogenesis (26).

### 3.3 Stromal cell domestication

After PMN has undergone the construction of an inflammatory environment and has a vascular supply of nutrients, stromal cells will also be domesticated by exosomes and become the cradle for future tumor cell landings, with the main cell groups including endothelial cells of blood vessels, epithelial cells of trachea or bronchi, and fibroblasts (44). It was demonstrated that the pro-tumorigenicity of miR-210 in A549 cells and their exosomes were increased upon induction of tissue inhibitor of metalloproteinase-1 (TIMP-1) tissue inhibitor, and that miR-210 accumulated in exosomes *in vitro* and *in vivo* after overexpression of TIMP-1 in tumor cells. These exosomes promote the formation activity of human umbilical vein endothelial cells (HUVECs), and these transformed stromal cells become paracrine to the malignancy, increasing angiogenesis (27). Exosomes from lung cancer similarly induce lung epithelial cells to exhibit immunomodulatory effects in lung homeostasis and mediate immunosuppressive PMN formation in lung metastases (28). Fibroblasts are a large class of stromal cell populations that can provide ample back-up for lung cancer cells. Circulating Lewis Lung Carcinoma (LLC)-derived exosome miR-3473b can be phagocytosed by lung fibroblasts, causing NF-κB activation in fibroblasts and increasing intrapulmonary colonization of lung tumor cells (29).

### 3.4 Bone marrow-derived cell domestication and recruitment

After building the basic material basis of PMN, metastatic tumor cells need to find pathway guides and escape from immune cells, and domesticating bone marrow-derived cells to achieve this goal is a “smart” choice for lung cancer cells, and macrophages and dendritic cells (DCs) seem to be more preferred. The macrophages in the tumor microenvironment are called tumor-associated macrophages (TAM) (45). In the NSCLC microenvironment, TAM constitute the major cellular component. They not only act as immunosuppressive cells, enabling NSCLC to immune evade, but also directly promote the proliferation, survival, invasion, and metastasis of cancer cells. TAM include both inflammatory or classically activated (M1) and anti-inflammatory or alternative activated (M2) phenotypes, while the M2 type of TAM has been shown to promote pre-metastatic ecology by secreting TGF-β, SDF-1 and VEGF through STAT3 signaling cascade site formation (46). During excessive primary tumor growth, hypoxic lung cancer cells promote macrophage M2 polarization and induce lung cancer progression by secreting exosomes that affect the PI3K/AKT signaling pathway *via* miR-21 (30). Another mechanism is that exosomes derived from lung cancer cells secrete TRIM59, which converts macrophages into tumor-promoting macrophages by regulating ABHD5 proteasome degradation, thereby activating the NLRP3 inflammatory vesicle signaling pathway and promoting lung cancer progression by secreting IL-1 (31). DC, as one of the most functional antigen-presenting cells, is necessary for lung cancer cells to achieve immune escape by remotely controlling DC the presence of EGFR in lung cancer exosomes induces tolerogenic DCs, which in turn induce the production of tumor antigen-specific regulatory T cells (Tregs), which suppress tumor antigen-specific CD8 T cells and induce immune tolerance in lung cancer patients (32). Exosomes from LLC lung cancer cells can block the differentiation of bone marrow progenitor cells into CD11c DCs and induce apoptosis at their source (33). In addition, exosomes derived from domesticated bone marrow cells can also suppress immunity and thus promote metastasis, e.g. exosomes from tumor-bearing mouse bone marrow-derived cells (BMDC) carry PD-L1, and PD-L1 on these exosomes is biologically functional and can inhibit CD8 T cell proliferation and activation *in vitro* and *in vivo* (34). Of course lung cancer cells can also exert immunosuppressive effects across bone marrow-derived cells, such as exosomes from lung cancer cells that express PD-L1 and promote tumor growth by reducing T cell activity to induce immune escape. The rationale is that exosomal PD-L1 inhibits T-cell secretion of interferon-γ (IFN-γ), reduces cytokine production and induces apoptosis of CD8 T cells to impair immune function and promote lung cancer metastasis (35).

### 3.5 Other factors affecting the formation of PMN

PMN formation is a sophisticated and complex project in which metabolic alterations, epithelial-mesenchymal transition (EMT) formation and even atmospheric pollutants may be involved, in addition to the standard processes involving inflammation, vasculature, stromal cells, and bone marrow-derived cells. Altered metabolism may be an important pathway in the differentiation of macrophages to an immunosuppressive phenotype by tumor-derived exosomes, such as increased PD-L1 expression through NF-κB-dependent, glycolysis-led metabolic reprogramming (36). Exosomes can be involved in the disruption of lipid metabolism in cancer cells and in the malignant progression of cancer (47). Exosomes derived from lung cancer cells promote lipid degradation in adipocytes, thereby providing energy for uncontrolled cell proliferation (37). In PMN, exosomes may be involved in EMT formation earlier (48), and exosomes have been shown to regulate many cellular functions by activating EMT and inducing pre-metastatic ecotone formation. Specific exosomal miRNAs, including miR-10aand miR-26a are involved in exosomal promotion of EMT in epithelial cells as well as migration and invasion of cancer cells, and may serve as novel biomarkers of the EMT process in lung cancer (38). Cancer-associated fibroblasts (CAF) may also be involved in the induction of EMT, such as SNAI1 delivered to recipient lung cancer cells *via* exosomes from CAF to promote EMT (39). As an organ in contact with the atmosphere, atmospheric particulate matter (PM) may be involved in the formation of PMN, as shown by a research team that injected LLC cells into C57BL/6 mice *via* tail vein and PM *via* nasal drip. The results showed that the lung metastasis foci were increased in the mice dripped with PM, indicating that PM promoted the lung metastasis of LLC cells. In addition, the expression levels of certain genes that promote tumor cell metastasis and promote tumor cell colonization at metastatic sites were significantly increased in the lungs of PM-exposed mice, and some of these genes were upregulated mainly in neutrophils, indicating that PM promotes the formation of pre-metastatic ecological sites in the lungs (40). This interesting finding may be an alternative strategy to reduce lung cancer incidence and improve prognosis in areas with harsh atmospheric conditions and a high prevalence of lung cancer.

## 4 Natural medicines intervene in the PMN formation link of lung cancer

We have learned above that exosomes are involved in PMN formation in lung cancer, and several reliable and important pathways have been summarized in the cumbersome PMN formation mechanism, and how to intervene in exosomes involved in these pathways may be the key to reduce lung cancer metastasis and improve the prognosis, the current therapeutic options for exosomes are more limited, such as Annette M. Marleau et al. using a AETHLONADPT plasma replacement technique, in which blood containing exosomes is filtered, found that targeted reduction of exosomes may become a treatment for lung cancer. In addition, ceramide may be one of the lipids required for exosome synthesis and its synthesis is mediated by neutral sphingomyelinase 2 (nSMase2). Therefore, the neutral inhibitor of nSMase2, GW4869, could be added to eventually eliminate or reduce exosome production (49). Modification of exosomes for antitumor is a popular direction and there are some promising advances, but there are still relatively few studies targeting exosomes to modulate PMN. Natural medicines have a huge number of potential options that can be explored if they are combined with TCM.

### 4.1 Regulation of tumor exosome secretion

The formation of PMN is closely related to the release of tumor exosomes, and the regulation of tumor exosome release is a key step, which is currently a hot direction in natural medicines research. Shikonin, a naphthoquinone isolated from Comfrey, has potent antitumor effects, and modulation of exosome miR-628-3p in non-small cell lung cancer cell lines has recently been shown to be a novel mechanism for its inhibition of lung cancer cell proliferation (50). Not only in lung cancer, comfrey can also inhibit MCF-7 cell proliferation by regulating exosomal miR-128 (51). Halofuginone, a small alkaloid extracted from the traditional Chinese herb Changshan, exerts anti-tumor effects by inhibiting the secretion and delivery of exosomal miR-31 through regulation of the HDAC2/cell cycle signaling axis, thereby inhibiting MCF-7 cell proliferation (52). Not only the single components of natural medicines, but also the combination of multiple natural medicines in traditional Chinese medicine can play the role of regulating exosomes. For example, Jinfu Kang preparation, which consists of 12 herbs, has been used in the clinical treatment of lung cancer for a long time, and it has been proved that Jinfu Kang can inhibit CTC-TJH-01 cells by negatively regulating the EGF signaling pathway in CTC-TJH-01 exosomes of lung cancer circulating tumor cells metastasis (53).

### 4.2 Intervention of inflammatory formation-related exosomes

From the above, we found that Toll-like receptor (TLR) may be an important inflammatory environment-forming molecule during PMN formation, and regulation of TLR-related pathways may be a reliable way to prevent the formation of pre-metastatic inflammatory environment. TLR is an important immune regulator for the regulation of intracellular inflammatory responses, and its downstream may be closely related to the activation of NOD-like receptor thermal protein domain associated protein 3 (NLRP3) (54). Expression of inflammatory vesicles NLRP3 also induces immunosuppressive effects such as M2 macrophages to promote tumor progression (55). Artemisinin (ART) is a classical antimalarial drug derived from the Chinese herb Artemisia annua for the treatment of autoimmune diseases with anti-inflammatory and immunomodulatory properties. New studies have shown that artemisinin can modulate inflammatory factors by mediating exosome release in human renal tubular epithelial cells (HK-2), which may be related to the inhibition of NF-κB/NLRP3 pathway by regulating exosome secretion (56). The same study showed that Zhen Wu Tang can regulate exosomal secretion to inhibit NF-kB/NLRP3 signaling pathway and thus control inflammatory diseases (57). Heat shock proteins are a key pathway for TLR initiation, and kaempferol, a potent flavonoid compound derived from cinnamon leaves, can reduce exosome secretion from cancer cells by regulating the protein expression of heat shock protein HSP 90-α (HSP90AA1), heat shock protein HSP 90-β (HSP90AB1), and ubiquitin-like modifier activator enzyme 1 (UBA1), vacuolar sorting protein 4(VPS4A) are important targets among them (58). TGF-β is also an important molecule in the inflammatory environment, and the combination of the multi-natural medicine Tongxinluo capsules can reduce the expression of TGF-β1-containing exosomes secreted by endothelial cells (59).

### 4.3 Intervention of angiogenesis-related exosomes

The study of exosomes related to intervention of tumor angiogenesis is not sufficient yet but has good potential for development. Steroidal glycoalkaloids from Solanum lyratum (Solanum lyratum Thunb.), a class of steroidal alkaloid glycosides with important antitumor activity, affect the formation of tumor exosomes, leading to changes in their functions, which in turn inhibit tumor angiogenesis and suppress the development of lung cancer A549 cells (60).

### 4.4 Intervention of bone marrow-derived cells to domesticate and recruit related exosomes

Modulation of bone marrow-derived cells seems to be another important direction for natural medicines to act, such as ginsenoside Rg1 by attenuating the release of macrophage-derived exosomes miR-21, which has been shown to be an important regulatory mechanism in lung cancer metastasis (61). Dahuang Zhechong Pill (DHZCP) significantly reduced serum exosomal CC chemokine ligand-2 (CCL2) levels and its receptor CCR2, which in exosomes triggers macrophage recruitment and converts M1/M2 macrophages in the liver to the M2 phenotype, and DHZCP modulated the pathway of CCL2 in exosomes reversing the pro-tumor polarization of macrophages (62). In addition to direct action on TAM-derived exosomes, some natural medicines may also act on TAM, the originator of CMPB90-1, a novel natural polysaccharide from Cordyceps sinensis, which translates immunosuppressive TAM by binding to toll-like receptor 2 (TLR2), polarizing TAM to M1 phenotype with antitumor effects and better safety profile. Studies in Chinese medicine compounds are also noteworthy (63).

### 4.5 Intervention of other pathways related to exosomes

Altered metabolism is a potentially potent player in PMN, and the active ingredient of the traditional Chinese medicine Comfrey is a naphthoquinone compound that inhibits glycolysis in non-small cell lung cancer cells by modulating the exosomal pyruvate kinase M2 pathway (64). EMT has also been published, and Jianshu Huayu Fang (includes ingredients such as *Salvia miltiorrhiza Bunge*, *Poria cocos* etc.) is a traditional Chinese medicinal preparation that can treat a wide range of malignancies, including a variety of malignancies, with antitumor effects that are partly EMT-mediated inhibition through exosome-mediated downregulation of intercellular miR-23a-3p transfer and subsequent blockade of Smad signaling, while suggesting that disruption of this exosomal miR-23a-3p/Smad signaling pathway may be an effective anti-tumor approach (65).

## 5 New interventions where natural medicines are viable

Natural medicines can modulate PMN through exosomes not only by modulating tumor-related exosomes, but also by using the properties of exosomes to achieve the role of modulating PMN-related mechanisms. In the current related research, using exosomes to encapsulate natural medicines seems to be a recommended option, and isolating their own exosomes from natural medicines for tumor treatment is also a feasible decision to explore the active ingredients of natural medicines. The isolation of exosomes from natural medicines for tumor treatment is also a feasible decision to explore the active ingredients of natural medicines (**Table 2**).

**Table 2:** Natural medicines that regulate the formation of PMN.

No	Mechanism	Natural medicines	Related exosomes	Function	Refs
1	Inhibition of exosome release	Shikonin	miR-628-3p	inhibits cell proliferation and induces apoptosis	(50)
		Shikonin	miR-128	inhibits cell proliferation	(51)
		Halofuginone	miR-31	regulates the level of cell cycle	(52)
		Jinfu Kang preparation (multiple natural medicines)	EGF	inhibits the metastasis	(53)
2	Inflammatory formation	ART	HK-2-derived exosomes	NF-κB/NLRP3	(56)
		Zhen Wu Tang (multiple natural medicines)	HK-2-derived exosomes	NF-κB/NLRP3	(57)
		kaempferol	VPS4A	HSP 90-α/HSP 90-β/UBA1	(58)
		Tongxinluo capsules (multiple natural medicines)	total exosomes from glomerular mesangial cells	TGF-β1	(59)
3	angiogenesis	Steroidal glycoalkaloids from Solanum lyratum	A549-derived exosomes	Lipid raft	(60)
4	Bone marrow-derived cell domestication and recruitment	ginsenoside Rg1	miR-21	macrophages	(61)
		DHZCP (multiple natural medicines)	CCL2	M2 TAM	(62)
		CMPB90-1	TLR2	M1 TAM	(63)
5	others	Shikonin	PKM2	inhibits glycolysis	(64)
		Jianshu Huayu Fang (multiple natural medicines)	miR-23a-3p	EMT	(65)

### 5.1 Exosomes loaded with natural medicines

Recently, several studies have shown that exosomes can be used as potential drug delivery system (DDS) in cancer therapy. With its small size, high stability, biocompatibility and safety profile, it can be used as an efficient tumor-targeting vehicle to deliver drugs to proximal or distal cells (66, 67). Exosomes derived from cancer cells are pro-trophic to their parental cells and can therefore be used as trojan horses to target these malignant cells (68). The ability of exosome biogenesis in different human cancer cells is positively correlated with the activation of STAT3/PKM2/SNAP23 pathway, and STAT3 plays a key role in regulating the biogenesis of tumor-derived exosomes (69). Curcumin has been shown to effectively inhibit STAT3 phosphorylation in small cell lung cancer by downregulating downstream regulatory proteins of STAT3, which contributes to the inhibition of cell proliferation, diminished cell colony formation, and reduced cell migration and invasion (70). For example, the anti-inflammatory activity of curcumin was improved when encapsulated in breast cancer cell-derived exosomes due to reduced off-target effects (71). Compared to free curcumin, exosome-encapsulated curcumin showed enhanced anti-lung cancer activity (72). The mechanism may be related to the modulation of exosomal TCF21, thereby inhibiting exosome-induced lung cancer metastasis (73). Exosomes from sources other than tumors also have enhanced effects on natural medicines components, such as norethindrone (NCTD), a derivative of zebularine isolated from the dried body of zebularine, which has multiple pharmacological activities including antitumor, and after using bone mesenchymal stem cell-derived exosomes (BMSC-Exos) were used as drug carriers to encapsulate NCTD, BMSC-Exos-NCTD significantly promoted cellular uptake and reduced cell migration and invasion of cancer (74). The surprise of the exosome encapsulation technology does not stop there, as milk-derived exosomes are equally effective, which provides sufficient carrier support for the prospective application of natural medicines after exosome encapsulation and greatly enhances the clinical feasibility. Celastrol (CEL) is a plant-derived triterpenoid derived from C. rhamnosus, a known inhibitor of Hsp90 and NF-κB activation pathways. milk-derived exosomes loaded with Celastrol (Exo-CEL) exhibited higher efficacy in inhibiting lung cancer cell proliferation *in vivo* and *in vitro* compared to free CEL (75).

### 5.2 Plant-derived exosomes

With advances in assay technology, the exploration of active ingredients of natural medicines has become increasingly sophisticated with a variety of potent monomers emerging, and the recent discovery of plant-derived exosomes for their antitumor effects provides an interesting angle for research. Citrus limon-derived nanovesicles inhibit cancer cell proliferation by inducing TRAIL-mediated cell death in human lung cancer cell line A549 cells and may have a potential role in inhibiting lung cancer metastasis (76). Ginger-derived exosomes were redesigned into nanocarriers designed to deliver adriamycin to colon tumor cells, and these nanocarriers effectively incorporated into and inhibited the growth of tumor model cells without the intrinsic properties of the encapsulated adriamycin exosomes likewise inducing significant antitumor effects by a mechanism that may be related to the regulation of pro-inflammatory cytokine levels (77).

## 6 Concluding remarks

Exosomes have been studied in tumors at all stages of tumor diagnosis, development, progression, and prognosis. miRNAs encapsulated in exosomes are not easily degraded, making them more suitable biomarker candidates, and thus the diagnostic value of blood-derived exosomal miRNA signatures has been extensively described in lung cancer and other types of cancers (78). Tumor relatively specific non-coding RNAs (miRNA, circRNA, lncRNA), double-stranded DNA and proteins have all been found to be present in exosomes, demonstrating their potential as diagnostic biomarkers (79). Then whether some exosomes can be used as specific tumor markers for lung cancer metastasis may be of interest. In this paper, based on the fact that the formation of PMN in lung cancer is necessary for the occurrence of metastasis, and that blocking this pathway is an important strategy to improve the prognosis of lung cancer patients in the future, we explored the role of exosomes in PMN formation in lung cancer, expecting to provide a reference for the discovery of specific tumor metastasis markers.

Natural medicines are limited by their complex composition, and relevant studies mostly stay on the inhibitory effect of a particular monomer on a certain tumor and the intervention of the corresponding pathway, thus *in vitro* experiments tend to be more than *in vivo* experiments, while clinical trials are even scarcer, limiting the application of this treasure trove. However, the complex composition of natural medicines may be more likely to play a role in this complex process of PMN formation, so the potential of natural medicines to intervene in the various pathways of exosome-mediated PMN formation is explored in this paper; unfortunately, relevant studies are still scarce, but there is still evidence available that suggests to us that natural medicines modulating exosomes have potential in PMN formation in lung cancer. Another situation in which natural medicines are limited by their application is their generally low anticancer activity, poor water solubility and poor absorption (80), and it would be beneficial to enhance these capabilities for the promotion of natural medicines. In this paper, we also found that exosome-encapsulated natural products significantly enhanced their antitumor effects and utilization, which is not a promising research direction.

Combining the regulatory role of natural medicines in lung cancer exosomes and the multi-faceted intervention role of PMN, we believe that there are several directions for further expansion of applications in the future clinic. Firstly, by engineering small cell exosomes to encapsulate natural medicines components, standardized production can also enhance their effects, promote new drug development, and make up for the shortage of tumor exosome-related therapeutic tools; secondly, tumor-secreted exosomes have different tissue and organophilic properties, and different kinds of natural medicines components have similar distribution relationships, which can be used precisely to stop metastasis after detecting exosomes of certain biomarkers. Thirdly, some natural medicines have been used in long-term anti-tumor use in a fixed ratio of synergistic effect, and for PMN formation process with complex mechanism, the combination of multiple natural medicines components may achieve better clinical efficacy. The exosomes of natural medicines themselves show us the rich value of natural medicines, which are promising in human efforts to continuously overcome tumor metastasis and improve the prognosis of tumor patients.

## Author contributions

JL designed the research study. X-YZ wrote the manuscript. All authors contributed to editorial changes in the manuscript. All authors read and approved the final manuscript.
